# *Syagrus coronata* fixed oil attenuates inflammation, oxidative stress and pulmonary alterations in L-arginine-induced pancreatitis

**DOI:** 10.1007/s10787-026-02300-8

**Published:** 2026-06-24

**Authors:** Bruna de Sousa Gomes, Beatriz Meyruze Barros da Fonsêca, Laís Ruanita Leopoldina Galvão, Beatriz Estandislau Lins de Carvalho, Paulo Henrique Eloi Fernandes, Edymilaís da Silva Sousa, Anderson Arnaldo da Silva, Júlio César Ribeiro dede Oliveira FariasAguiar, Daniela Maria do Amaral Ferraz Navarro, Alisson Macário de Oliveira, Wêndeo Kennedy Costa, Márcia Vanusa da Silva, Maria Tereza dos Santos Correia

**Affiliations:** 1 Departamento de Bioquímica, Universidade Federal de Pernambuco, Recife, PE 50670-901 Brazil; 2 Departamento de Química Fundamental, Universidade Federal de Pernambuco, Recife, PE 50670-901 Brazil; 3 Departamento de Anatomia, Universidade Federal de Pernambuco, Recife, PE 50670-901 Brazil; 4 Departamento de Farmácia, Universidade Federal do Rio Grande do Norte, Natal, RN 59012-570 Brazil

**Keywords:** Acute pancreatitis, Cytokines, Oxidative stress, Lung injury, *Syagrus coronata* fixed oil

## Abstract

Acute pancreatitis is an inflammatory condition characterized by an intense systemic inflammatory response and increased oxidative stress, which may compromise distant organs such as the lungs. In this context, natural compounds with antioxidant and anti-inflammatory properties have been investigated as potential therapeutic agents. Therefore, this study aimed to evaluate the effect of the fixed oil from *Syagrus coronata* in an experimental model of L-arginine-induced acute pancreatitis. Pancreatitis was induced in mice by two intraperitoneal injections of L-arginine (8%, 4 g/kg). Animals were orally treated with *Syagrus coronata* fixed oil (ScFO) at doses of 25, 50, or 100 mg/kg after disease induction. Serum biochemical parameters (amylase, lipase, and glucose), inflammatory cytokines (IL-1β, IL-6, and TNF-α), oxidative stress markers in lung tissue (MDA, SOD, and CAT), and histological alterations were evaluated. Pancreatitis induction significantly increased amylase, lipase, glucose, inflammatory cytokines, and lipid peroxidation, while reducing antioxidant enzyme activity in lung tissue. Treatment with ScFO at 50 mg/kg reduced amylase (35.12%), lipase (40.74%), and glucose levels (20.24%), whereas the 100 mg/kg dose produced reductions of 56.44%, 59.26%, and 30.95%, respectively. Decreases in IL-1β (32.05% and 52.56%), IL-6 (31.34% and 52.99%), and TNF-α (29.55% and 50%) were also observed at doses of 50 and 100 mg/kg. In lung tissue, the oil reduced MDA levels by 34.78% and 54.71% and increased SOD and CAT activity at higher doses. The 25 mg/kg dose showed no significant effect. These findings indicate that ScFO exerts a protective effect in experimental pancreatitis by reducing systemic inflammation and oxidative stress, particularly at doses of 50 and 100 mg/kg.

## Introduction

Acute pancreatitis is characterized by a sudden inflammatory process of the pancreas, clinically manifested by severe abdominal pain, elevated serum pancreatic enzymes, and potentially rapid disease progression, often associated with significant morbidity (Garber et al. 2018; Van den Berg and Boermeester 2023). Globally, its estimated incidence ranges from 30 to 40 cases per 100,000 inhabitants per year, making it one of the leading causes of hospital admission for gastrointestinal disorders (Petrov and Yadav 2019).

Several etiological factors are involved in the development of acute pancreatitis, particularly biliary obstruction, excessive alcohol consumption, metabolic disorders, and endoscopic procedures, especially endoscopic retrograde cholangiopancreatography (Lee and Papachristou 2019; Boxhoorn et al. 2020; Yang and McNabb-Baltar 2020). Regardless of the triggering factor, the pathophysiology of the disease involves injury to pancreatic acinar cells and the premature intrapancreatic activation of digestive enzymes. This initial event triggers both local and systemic inflammatory responses, characterized by the recruitment of immune cells and the release of pro-inflammatory mediators. When this inflammatory process is not adequately controlled, severe complications may occur, including pancreatic necrosis, secondary infection, and multiple organ failure (Waller et al. 2018; Habtezion et al. 2019; Boxhoorn et al. 2020).

Current clinical management of acute pancreatitis is mainly based on supportive care, including adequate fluid resuscitation, pain control, oxygen therapy, and appropriate nutritional strategies (Gliem et al. 2021). Despite advances in intensive care and risk stratification, no specific pharmacological therapies are currently available to directly modulate pancreatic inflammation, highlighting the need to explore new therapeutic approaches (He et al. 2025).

In this context, natural compounds with traditional medicinal use and recognized anti-inflammatory properties have attracted increasing scientific interest as potential therapeutic agents. *Syagrus coronata* (Mart.) Becc. (Arecaceae), popularly known as licuri, ouricuri, or coquinho, is a palm species native to the Brazilian semi-arid region and the Caatinga biome, with well-documented ethnopharmacological relevance (Drumond 2007; Oliveira de Souza et al. 2017). The fixed oil extracted from its fruits is rich in fatty acids, conferring significant nutritional and pharmacological value, and has been associated with antibacterial and antifungal activities (Souza dos Santos et al. 2019; Cintra et al. 2025), as well as anti-inflammatory and antinociceptive effects (Barbosa et al. 2024; Oliveira Alves et al. 2024), in addition to metabolic and cardiovascular benefits (Silva et al. 2026).

Despite these promising properties, the effects of *S. coronata* fixed oil (ScFO) on pancreatic inflammation have not yet been investigated. Therefore, the present study aimed to evaluate the impact of ScFO in an experimental model of acute pancreatitis by analyzing inflammatory biomarkers, oxidative stress parameters, and potential mechanisms underlying its biological activity.

## Materials and methods

### Plant material

Kernels of *Syagrus coronata* (Mart.) Becc. (http://www.theplantlist.org/tpl1.1/record/kew-198799) were obtained from the Caatinga region, specifically in the municipality of Capim Grosso, Bahia, Brazil (11° 22′ 54″ S, 40° 00′ 46″ W). A voucher specimen was deposited at the Professor Vasconcelos Sobrinho Herbarium of the Federal Rural University of Pernambuco (UFRPE) under accession number PEUFR 55,147. The fixed oil was extracted from the kernels using a cold electric press, yielding a yellowish viscous oil referred to as *Syagrus coronata* fixed oil (ScFO).

### Determination of fatty acid profile by acid esterification and gas chromatography

Fatty acids present in the oil were converted into fatty acid methyl esters (FAMEs) by acid-catalyzed esterification using BF_3_ in methanol, according to ISO method 5509 (1978), with minor modifications. Briefly, approximately 150 mg of oil was dissolved in 2 mL of chloroform, followed by the addition of 2 mL of 0.5 mol L^−1^ NaOH in methanol. The mixture was heated in a water bath at 100 °C for 5 min. Subsequently, 2 mL of BF_3_ in methanol (1.3 M) was added under an inert atmosphere, and the reaction mixture was maintained under reflux for 30 min.

After cooling, the mixture was transferred to a separatory funnel, and 5 mL of heptane and 0.5 mL of saturated NaCl solution were added. Following vigorous shaking and phase separation, the upper organic phase was collected, dried over anhydrous sodium sulfate, and filtered. The solvent was evaporated under a nitrogen stream, and the resulting sample was used for chromatographic analysis.

FAME identification was performed by gas chromatography coupled to mass spectrometry (GC–MS) using an Agilent 5975C Series system (Agilent Technologies, Palo Alto, USA) equipped with a DB-5 capillary column (30 m × 0.25 mm; 0.25 µm film thickness). The oven temperature program was initially set at 60 °C for 2 min, followed by heating at 3 °C min^−1^ up to 260 °C (held for 40 min), and subsequently increased at 4 °C min^−1^ to 260 °C and maintained for 7.5 min. Helium was used as the carrier gas at a flow rate of 1.0 mL min^−1^. The injector temperature was set at 230 °C and the interface temperature at 260 °C. The mass spectrometer operated in scan mode (35–550 m/z) with an ionization energy of 70 eV. Compound identification was performed by comparing the obtained mass spectra with those available in the NIST and Wiley libraries, as well as by comparison with authentic FAME standards (C4–C24).

Quantification of fatty acids was carried out by gas chromatography with flame ionization detection (GC-FID) using a TRACE GC Ultra system (Thermo Scientific) equipped with a VB-5 column (30 m × 0.25 mm; 0.25 µm film thickness). Nitrogen was used as the carrier gas at a flow rate of 1 mL min^−1^. The oven temperature program was identical to that used for GC–MS analysis, with injector and detector temperatures set at 230 °C and 260 °C, respectively. All analyses were performed in triplicate, and the results were expressed as mean ± standard deviation.

### Ethical considerations and animal housing

This study was submitted to and approved by the Animal Ethics Committee of the Federal University of Pernambuco (CEUA/UFPE) under protocol number 0012/2025. All experimental procedures were conducted in strict accordance with Brazilian legislation governing the use of animals in scientific research (Law No. 11,794/2008).

Thirty male Swiss mice weighing 30–40 g (approximately 60 days old) were obtained from the Animal Facility Center (CEBIO) of UFPE. The animals were housed under standard laboratory conditions with a 12 h light/dark cycle, controlled temperature (22 ± 2 °C), and relative humidity of 50–55%. Animals had free access to water and standard rodent chow ad libitum, following the recommendations of the *Guide for the Care and Use of Laboratory Animals* (National Research Council 2011).

### L-arginine–induced acute pancreatitis model

Mice were randomly distributed into five experimental groups (n = 6 per group). The Sham group received only 0.9% saline solution via intraperitoneal (i.p.) injection, followed by oral administration of saline solution. The disease control group received two i.p. injections of L-arginine (8% solution, 4 g/kg, pH 7.0) with a one-hour interval between administrations, followed by oral treatment with 0.9% saline solution (Kui et al. 2015; Ohkawara et al. 2017; Ono et al. 2023).

The remaining three treatment groups received L-arginine under the same conditions as the disease control group but were orally treated with ScFO at doses of 25, 50, or 100 mg/kg, respectively, at 24, 48, and 72 h after pancreatitis induction. All oral treatments were administered by gavage in a volume of 10 mL/kg of body weight. Seventy-two hours after induction, the animals were euthanized using a lethal dose of ketamine (300 mg/kg) and xylazine (30 mg/kg) administered intraperitoneally, as recommended by the guidelines of the American Veterinary Medical Association (AVMA 2020). Immediately after euthanasia, 1 mL of blood was collected by cardiac puncture from all groups for biochemical and inflammatory cytokine analyses. Lung tissue samples were also collected for histological examination and oxidative stress analysis.

### Serum biochemical assessment of pancreatic function

Blood samples were transferred to tubes without anticoagulant and allowed to clot at room temperature for approximately 30 min. The tubes were then centrifuged at 3000 rpm for 10 min to obtain serum, which was carefully separated and stored at − 20 °C until biochemical analysis.

Serum activities of amylase and lipase, used as markers of pancreatic injury, were determined by colorimetric methods using commercial diagnostic kits (Labtest Diagnóstica S.A., Lagoa Santa, MG, Brazil), strictly following the manufacturer’s instructions. Reaction readings were performed using an automated spectrophotometer, and the results were expressed in international units per liter (U/L). Serum glucose levels were determined using the enzymatic glucose oxidase–peroxidase method (GOD-POD) with a commercial kit (Labtest Diagnóstica S.A., Lagoa Santa, MG, Brazil). Absorbance was measured spectrophotometrically at a wavelength of 505 nm, and the results were expressed in mg/dL.

### Quantification of serum cytokines

Serum levels of tumor necrosis factor-alpha (TNF-α), interleukin-1 beta (IL-1β), and interleukin-6 (IL-6) were determined using enzyme-linked immunosorbent assay (ELISA) kits (eBioscience, USA) according to the manufacturer’s instructions. Briefly, 96-well plates were coated with capture antibodies specific for each cytokine and incubated overnight at 4 °C. After washing steps, the plates were blocked and then loaded with standard curves and test samples. Following overnight incubation at 4 °C, the plates were washed with wash buffer, and biotinylated detection antibodies were added, followed by incubation at room temperature for 1 h. 

After repeated washing, the enzyme conjugate was added, and the plates were incubated for an additional 30 min at room temperature. The plates were washed again, and tetramethylbenzidine (TMB) substrate was added, followed by incubation for 15 min at room temperature in the dark. The reaction was stopped by adding the stop solution, and optical density was measured at 450 nm using a microplate ELISA reader (BioTek Instruments uQUANT MQX200 microplate spectrophotometer). Cytokine concentrations were calculated based on the standard curve generated for each analyte. The ELISA technique is widely used for cytokine quantification in experimental models of pancreatitis (Sendler et al. 2018).

### Histological processing

Lung tissue was surgically excised, and the specimens were fixed for 48 h in 10% buffered formalin in phosphate-buffered saline (PBS; pH 7.2). The samples were subsequently washed under running water, dehydrated in increasing concentrations of ethanol (70%, 80%, 90%, and absolute), cleared in xylene, and embedded in histological paraffin.

Tissue Sects. (5 µm thick) were obtained using a Leica RM2125RT manual rotary microtome. The sections were mounted on glass slides and stained using the standard hematoxylin and eosin (H&E) technique. Microscopic examination was performed under a Zeiss Primostar 3 bright-field optical microscope, and images were captured using a Zeiss ERc5s digital camera at 200 × magnification.

Histopathological analysis was conducted through bright-field microscopy, with emphasis on identifying histological alterations related to disturbances in cellular growth and differentiation, inflammatory processes, and hemodynamic alterations. The results were presented descriptively (Fischer et al. 2008; Matute-Bello et al. 2011).

### Oxidative stress analysis

One lobe of lung tissue (500 mg) from each animal was surgically removed and homogenized in 50 mM Tris–HCl buffer (pH 7.4) containing 1 mM EDTA, 1 mM sodium orthovanadate, and 2 mM phenylmethylsulfonyl fluoride. The homogenate was centrifuged at 2500 rpm for 10 min at 4 °C, and the supernatant was collected for oxidative stress analyses.

Total protein content was determined using the Bradford method (1976). Lipid peroxidation was assessed by measuring thiobarbituric acid reactive substances (TBARS), with results expressed as nmol of malondialdehyde (MDA) per mg of protein (Ohkawa et al. 1979). This method is widely considered a gold-standard approach for evaluating oxidative lipid damage (Tsikas 2017). Superoxide dismutase (SOD) activity was measured by monitoring the inhibition kinetics of epinephrine auto-oxidation at 480 nm (Misra and Fridovich 1972), with enzymatic activity expressed as U/mg of protein. Catalase (CAT) activity was determined by monitoring the decrease in absorbance at 240 nm between the first and sixth minutes of the reaction, with enzymatic activity expressed as mU/mg of protein (Beers and Sizer 1952).

### Statistical analysis

Data are presented as mean ± standard deviation for each experimental group. Statistical comparisons were performed using one-way analysis of variance (ANOVA) followed by Tukey’s post hoc test for multiple comparisons. All statistical analyses were conducted using GraphPad Prism version 8 (GraphPad Software, San Diego, CA, USA), and statistical significance was established at *p* < 0.05.

## Results and discussion

The fatty acid composition of *Syagrus coronata* fixed oil was determined by gas chromatography following derivatization to fatty acid methyl esters (FAMEs). Compound identification was performed by comparing the retention times of the sample with those of the corresponding analytical standards. Eight fatty acids were identified, including caprylic (C8:0), capric (C10:0), lauric (C12:0), myristic (C14:0), palmitic (C16:0), stearic (C18:0), oleic (C18:1), and linoleic (C18:2) acids. Lauric acid (C12:0) was the major component of the oil, accounting for 45.80% of the total composition, followed by myristic (13.60%), oleic (13.24%), and caprylic acids (11.18%).

Overall, saturated fatty acids predominated (85.36%), whereas unsaturated fatty acids accounted for 14.64% of the total composition. The total percentage of identified fatty acids corresponded to 100% of the analyzed oil.

The lipid profile observed for *S. coronata* fixed oil reveals a marked predominance of saturated fatty acids, particularly lauric acid, which accounted for nearly half of the total composition. This pattern is characteristic of several species within the Arecaceae family, whose oils typically contain high concentrations of medium-chain fatty acids such as caprylic, capric, and lauric acids (Santos Souza et al. 2021; Ibiapina et al. 2022; Sales et al. 2023; Araújo et al. 2025).

The high proportion of lauric acid (C12:0) has also been reported in other studies involving *S. coronata* (Santos Souza et al. 2021; Barbosa et al. 2024). This finding is particularly relevant from a biological perspective, as this fatty acid exhibits well-documented antimicrobial (Souza dos Santos et al. 2019), anti-inflammatory (Barbosa et al. 2024; Silva et al. 2026), and antioxidant properties (Oliveira Alves et al. 2024), which may contribute to the pharmacological effects observed in experimental studies involving palm oils. In addition, medium-chain fatty acids are rapidly metabolized and play an important role in the modulation of inflammatory processes and in protection against oxidative damage in tissues (Pereira et al. 2023).

The oil also contained relevant amounts of myristic acid (C14:0) and oleic acid (C18:1). Oleic acid, a monounsaturated fatty acid widely found in vegetable oils, has been associated with cardioprotective, antioxidant, and anti-inflammatory properties and may contribute to the biological effects of the oil (Jagannathan et al. 2020; Smith et al. 2020; Leon-Aparicio et al. 2022; Santa-María et al. 2023). Although unsaturated fatty acids were present in lower proportions, mainly represented by oleic and linoleic acids, these compounds play an important role in maintaining cell membrane stability and modulating inflammatory pathways (Nava Lauson et al. 2023; Wang et al. 2024).

Overall, the fatty acid profile observed suggests that *S. coronata* oil presents a typical composition of oils rich in medium-chain fatty acids, a characteristic that may be associated with its antioxidant, anti-inflammatory, and tissue-protective potential, reinforcing its relevance for pharmacological and biotechnological applications. Based on these findings, the effect of *S. coronata* fixed oil was subsequently evaluated in an experimental model of L-arginine-induced acute pancreatitis in mice.

This experimental model is widely used to investigate pathophysiological mechanisms and potential therapeutic agents, as it reproduces key features of the disease, including acinar cell damage, increased circulating pancreatic enzymes, and a systemic inflammatory response (Khurana et al. 2023; Ono et al. 2023). Thus, serum biochemical parameters related to pancreatic function were evaluated to determine the possible protective effect of the oil against experimentally induced pancreatic injury.

Serum biochemical analysis was performed to assess pancreatic function and the protective effect of ScFO in the L-arginine–induced acute pancreatitis model. As expected, the disease control group (L-arginine) showed a significant increase in serum levels of amylase, lipase, and glucose compared with the Sham group, confirming the effective induction of pancreatitis.

Treatment with ScFO produced a dose-dependent effect on the evaluated parameters. The 25 mg/kg dose did not significantly reduce serum levels of pancreatic enzymes or glucose, presenting values similar to those observed in the disease control group. In contrast, animals treated with 50 mg/kg of ScFO exhibited a significant reduction in amylase (35.12%), lipase (40.74%), and glucose levels (20.24%) compared with the L-arginine group. Even more pronounced effects were observed with the 100 mg/kg dose of ScFO, which resulted in reductions of 56.44% in amylase, 59.26% in lipase, and 30.95% in glucose levels, approaching the values observed in the Sham group.

These findings suggest that ScFO exerts a significant protective effect against L-arginine–induced pancreatic damage, particularly at higher doses, indicating a clear dose–response relationship in the evaluated experimental model. Interestingly, the absence of significant protective effects at the 25 mg/kg dose reinforces the specificity of the pharmacological response and suggests the existence of a minimum effective therapeutic threshold for ScFO activity in experimental pancreatitis.

The results obtained demonstrate that L-arginine administration significantly increased serum levels of amylase and lipase, confirming the successful induction of acute pancreatitis and the impairment of pancreatic acinar cell integrity. This increase is associated with the release of digestive enzymes into the systemic circulation as a consequence of cellular damage and structural disorganization of the pancreas. In addition, the elevated glucose levels observed in the disease control group suggest dysfunction of pancreatic β-cells, indicating that the experimental model also compromises the endocrine function of the organ. This finding is consistent with previous reports describing L-arginine–induced pancreatitis as a model capable of producing both exocrine pancreatic injury and systemic metabolic disturbances (Goodarzi et al. 2021; Hart et al. 2021; Ono et al. 2023) (Tables 1, 2).

**Table 1:** Fatty acid profile of *Syagrus coronata* fixed oil determined by gas chromatography

Compounds	Retention time			Symbol	%	SD
Methyl ester	Fatty acid	Sample	Standard			
Methyl caprylate	Caprylic acid	15.65	15.55	C8:0	11,18	0,12
Methyl caprate	Capric acid	24.13	24.13	C10:0	6,94	0,06
Methyl laurate	Lauric acid	32.34	32.10	C12:0	45,80	0,30
Methyl myristate	Myristic acid	39.41	39.35	C14:0	13,60	0,21
Methyl palmitate	Palmitic acid	45.92	46.00	C16:0	6,30	0,06
Methyl linoleate	Linoleic acid	50.88	50.84	C18:2	1,40	0,04
Methyl oleate	Oleic acid	51.23	51.18	C18:1	13,24	0,22
Methyl stearate	Stearic acid	51.87	52.02	C18:0	1,54	0,02
Unsaturated fatty acids					14.64	
Saturated fatty acids:					85.36	
Total identified fatty acids					100.00

**Table 2:** Effect of *Syagrus coronata* fixed oil (ScFO) on serum biochemical parameters in an L-arginine–induced pancreatitis model

Groups	Amylase (U/L)	Lipase (U/L)	Glucose (mg/dL)
Sham	612 ± 54	48 ± 6	102 ± 8
L-arginine	1825 ± 136*	162 ± 14*	168 ± 12*
ScFO 25 mg/kg	1768 ± 128*	154 ± 13*	162 ± 11*
ScFO 50 mg/kg	1184 ± 102*#	96 ± 9*#	134 ± 10*#
ScFO 100 mg/kg	795 ± 74*#	66 ± 7*#	116 ± 9*#

Values are expressed as mean ± standard deviation (SD) (n = 6). Statistical analysis was performed using one-way ANOVA followed by Tukey’s post hoc test. *p < 0.05 compared with the Sham group; #*p* < 0.05 compared with the L-arginine group

In experimental models of pancreatic injury, increased serum concentrations of amylase and lipase are associated with acinar cell disruption, resulting in the leakage of these digestive enzymes into the bloodstream. Conversely, substances with antioxidant or cytoprotective properties may contribute to the preservation of cellular membrane integrity, thereby reducing this process (Tenner et al. 2024).

Studies conducted by Bauer et al. (2013), Souza et al. (2021), and Toledo (2024) indicate that this oil is predominantly composed of fatty acids such as lauric, caprylic, and myristic acids, in addition to compounds with antioxidant activity. These characteristics have been associated with cellular protection and the maintenance of membrane stability. Such compounds may modulate inflammatory pathways by inhibiting pro-inflammatory mediators and reducing the production of reactive oxygen species, thereby contributing to the attenuation of pancreatic damage (Barbosa et al. 2024; Oliveira Alves et al. 2024; Santos Nunes et al. 2024).

The systemic inflammatory response associated with acute pancreatitis was evaluated by measuring the pro-inflammatory cytokines IL-1β, IL-6, and TNF-α. As shown in Table 3, pancreatitis induction significantly increased the levels of these cytokines compared with the Sham group. The pancreatitis group presented IL-1β (312 pg/mL), IL-6 (268 pg/mL), and TNF-α (352 pg/mL), corresponding to approximate increases of 254%, 272%, and 203%, respectively, relative to the Sham group.

**Table 3:** Effect of *Syagrus coronata* fixed oil (ScFO) on serum levels of pro-inflammatory cytokines in an experimental model of L-arginine–induced pancreatitis

Groups	IL-1β (pg/mL)	IL-6 (pg/mL)	TNF-α (pg/mL)
Sham	88 ± 3	72 ± 4	116 ± 3
L-arginine	312 ± 12*	268 ± 10*	352 ± 14*
ScFO 25 mg/kg + L-arginine	298 ± 11*	255 ± 9*	336 ± 12*
ScFO 50 mg/kg + L-arginine	212 ± 9*#	184 ± 8*#	248 ± 10*#
ScFO 100 mg/kg + L-arginine	148 ± 7*#	126 ± 6*#	176 ± 8*#

Values are expressed as mean ± standard deviation (SD) (n = 6). Statistical analysis was performed using one-way ANOVA followed by Tukey’s post hoc test. *p < 0.05 compared with the Sham group; #*p* < 0.05 compared with the L-arginine group

Treatment with ScFO demonstrated a dose-dependent anti-inflammatory effect. The 25 mg/kg dose did not produce a significant reduction in inflammatory cytokines, showing values similar to those observed in the pancreatitis group. In contrast, treatment with 50 mg/kg significantly reduced IL-1β, IL-6, and TNF-α levels, corresponding to decreases of approximately 32.05%, 31.34%, and 29.55%, respectively, compared with the pancreatitis group. The highest tested dose (100 mg/kg) produced the most pronounced effect, reducing IL-1β, IL-6, and TNF-α levels by approximately 52.56%, 52.99%, and 50%, respectively, approaching the levels observed in the Sham group. These findings suggest that ScFO exerts a systemic anti-inflammatory effect by reducing the production of pro-inflammatory cytokines associated with pancreatic inflammation.

Acute pancreatitis is characterized by a pronounced systemic inflammatory response, primarily mediated by the release of pro-inflammatory cytokines, including IL-1β, IL-6, and TNF-α, which play a central role in the progression of pancreatic injury and amplification of the inflammatory cascade (Hoque et al. 2014; Li et al. 2015; Caronni et al. 2023). TNF-α is one of the earliest cytokines released during pancreatitis and contributes to leukocyte activation and the induction of other inflammatory mediators (Qi-Xiang et al. 2022; Peng et al. 2024). Similarly, IL-1β contributes to the amplification of the inflammatory response and promotes the recruitment of immune cells to the site of injury (Li et al. 2015; Caronni et al. 2023). IL-6, in turn, has been associated with the progression of systemic inflammation and the severity of pancreatitis (Peng et al. 2024; Liu et al. 2025).

The reduction observed in inflammatory cytokines may be associated with the chemical composition of the oil, characterized by the presence of fatty acids with inflammation-modulating properties. These compounds may inhibit the activation of cellular inflammatory pathways, thereby reducing the production of pro-inflammatory mediators and attenuating pancreatic injury. Medium-chain fatty acids, particularly lauric acid, have been reported to modulate intracellular inflammatory signaling pathways, including inhibition of NF-κB activation, resulting in reduced transcription of pro-inflammatory cytokines such as TNF-α, IL-1β, and IL-6. In parallel, antioxidant effects may also involve activation of Nrf2-dependent pathways, which regulate the expression of endogenous antioxidant enzymes and contribute to cellular protection against oxidative stress (Barbosa et al. 2024; Oliveira Alves et al. 2024).

Taken together, these findings suggest that ScFO has the potential to modulate the inflammatory response associated with acute pancreatitis, contributing to the reduction of tissue damage and improvement of the systemic inflammatory state.

**Fig. 1:**
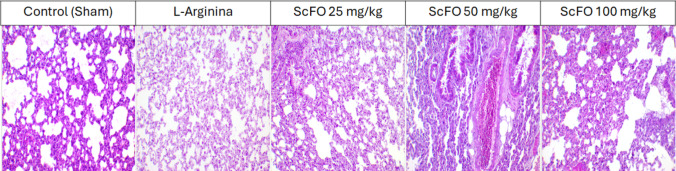
Lung histology in L-arginine–induced pancreatitis following treatment with *Syagrus coronata* fixed oil (ScFO). Representative photomicrographs of lung tissue from the Sham, L-arginine, and *Syagrus coronata*–treated groups (25, 50, and 100 mg/kg). Sections were stained with hematoxylin and eosin (H&E) and observed at an original magnification of 200 ×

Histological analysis of lung tissue was performed to investigate possible morphological alterations associated with experimental pancreatitis and to evaluate the effect of ScFO treatment. As illustrated in Fig. 1, the Sham group exhibited relatively preserved pulmonary architecture, with visible alveolar chambers and mild epithelial thickening, as well as small amounts of secretion within some alveolar spaces. In the L-arginine group, alterations in the organization of alveolar structures were observed, mainly characterized by changes in alveolar chamber morphology and modification of the thickness of the respiratory epithelium.

In animals treated with ScFO at the dose of 25 mg/kg, the histological characteristics were similar to those observed in the disease control group, with persistent alterations in alveolar chamber morphology associated with mildly thickened epithelium and the presence of secretion in some alveolar spaces. In contrast, the group treated with 50 mg/kg of ScFO exhibited morphological alterations in alveolar chambers accompanied by epithelial thickening and signs of vascular congestion. In addition, the presence of blood cells was observed within the lumen of bronchi and bronchioles. Animals treated with 100 mg/kg of ScFO still showed morphological alterations in the alveolar chambers; however, the respiratory epithelium appeared less thickened compared with the other experimental groups, suggesting a trend toward structural preservation of lung tissue.

The pulmonary alterations observed in the present study are consistent with systemic manifestations commonly associated with experimental acute pancreatitis. Pancreatic inflammation may trigger a systemic inflammatory response capable of affecting distant organs, including the lungs, promoting alterations in alveolar architecture, changes in respiratory epithelial thickness, and vascular disturbances (Hu et al. 2023; Liu et al. 2024; Peng et al. 2024).

In the present study, the L-arginine group exhibited morphological alterations in alveolar chambers and changes in epithelial thickness. These findings may be associated with the systemic inflammatory response previously evidenced by increased levels of the pro-inflammatory cytokines IL-1β, IL-6, and TNF-α. These cytokines play an important role in amplifying inflammation and may contribute to structural alterations in extra-pancreatic tissues, including the lungs (Hu et al. 2023; Wiley et al. 2023; Kınacı et al. 2024).

Administration of ScFO demonstrated distinct effects depending on the dose used. The 25 mg/kg dose presented histological characteristics similar to those observed in the L-arginine group, which is consistent with the previously described biochemical and inflammatory findings, in which this dose did not produce significant reductions in inflammatory markers or pancreatic injury. In contrast, higher doses, particularly 100 mg/kg, showed a tendency toward reduced epithelial thickening in alveolar structures, suggesting a possible modulation of structural alterations associated with systemic inflammation.

These findings may be related to the chemical composition of ScFO, which is characterized by the predominance of medium-chain fatty acids, particularly lauric acid, as well as fatty acids such as myristic, oleic, and caprylic acids. These compounds have been described in the literature as possessing anti-inflammatory, antioxidant, and immunomodulatory properties, which may contribute to the attenuation of systemic inflammatory processes (Gaete et al. 2023; Kaminskas et al. 2025; Wang et al. 2025). Fatty acids such as oleic acid, for example, have been associated with the modulation of cellular inflammatory pathways, while medium-chain fatty acids may influence metabolic and inflammatory processes, favoring the reduction of pro-inflammatory mediator production (Santa-María et al. 2023; Lee et al. 2024).

Thus, the tendency toward reduced epithelial thickness observed in the group treated with 100 mg/kg of ScFO may reflect a possible modulation of the systemic inflammatory response induced by pancreatitis. This result is consistent with the previous findings of this study, in which higher doses of the oil significantly reduced inflammatory cytokines and biochemical markers of pancreatic injury. Taken together, these results reinforce the hypothesis that ScFO may exert a modulatory effect on systemic inflammatory processes associated with experimental pancreatitis.

Oxidative stress in lung tissue was evaluated by determining malondialdehyde (MDA) levels, a marker of lipid peroxidation, as well as the activities of the antioxidant enzymes superoxide dismutase (SOD) and catalase (CAT). As shown in Table 4, induction of pancreatitis by L-arginine significantly increased MDA levels in lung tissue, accompanied by a reduction in the activities of the antioxidant enzymes SOD and CAT compared with the Sham group, indicating increased pulmonary oxidative damage associated with the systemic inflammatory response.

**Table 4:** Effect of *Syagrus coronata* fixed oil (ScFO) on oxidative stress markers in lung tissue in an experimental model of L-arginine–induced pancreatitis

Groups	MDA	SOD	CAT
Sham	2.13 ± 0.28	9.42 ± 0.81	7.86 ± 0.72
L-arginine	6.47 ± 0.63*	4.31 ± 0.47*	3.89 ± 0.41*
ScFO 25 mg/kg	6.12 ± 0.58*	4.53 ± 0.49*	4.06 ± 0.43*
ScFO 50 mg/kg	4.22 ± 0.41*#	5.82 ± 0.61*#	5.16 ± 0.54*#
ScFO 100 mg/kg	2.93 ± 0.33*#	6.91 ± 0.69*#	6.16 ± 0.63*#

MDA: malondialdehyde (nmol/mg protein); SOD: superoxide dismutase (U/mg protein); CAT: catalase (U/mg protein). Values are expressed as mean ± standard deviation (SD) (n = 6). Statistical analysis was performed using one-way ANOVA followed by Tukey’s post hoc test. *p < 0.05 compared with the Sham group; #*p* < 0.05 compared with the L-arginine group

Treatment with ScFO showed a dose-dependent effect on the evaluated parameters. Administration of 25 mg/kg did not produce significant changes in MDA, SOD, or CAT levels compared with the L-arginine group. In contrast, the groups treated with 50 mg/kg and 100 mg/kg exhibited a significant reduction in lipid peroxidation and increased activity of antioxidant enzymes. Relative to the L-arginine group, treatment with 50 mg/kg of ScFO reduced MDA levels by 34.78%, while the 100 mg/kg dose produced a reduction of 54.71%. In parallel, an increase in antioxidant enzyme activity was observed, with SOD activity rising by 35.03% and 60.32%, and CAT activity increasing by 32.65% and 58.35% at doses of 50 mg/kg and 100 mg/kg, respectively. These results suggest that ScFO contributes to the modulation of oxidative stress in lung tissue.

Oxidative stress plays an important role in the systemic complications associated with acute pancreatitis. Pancreatic inflammation may trigger a systemic inflammatory response characterized by the release of pro-inflammatory mediators and reactive oxygen species, which contribute to oxidative damage in extra-pancreatic tissues (Kong et al. 2021; Xia et al. 2024; Chen et al. 2025).

In the present study, induction of pancreatitis by L-arginine significantly increased MDA levels in lung tissue, indicating enhanced lipid peroxidation. Simultaneously, a reduction in the activities of the antioxidant enzymes SOD and CAT was observed, suggesting impairment of the pulmonary antioxidant defense system. These findings are consistent with previous reports describing oxidative stress as one of the major mechanisms involved in lung injury associated with experimental pancreatitis (Kong et al. 2021; Jin et al. 2023; Liu et al. 2023).

These effects may be related to the chemical composition of ScFO, which is characterized by a predominance of medium-chain fatty acids, particularly lauric acid, as well as other fatty acids such as myristic, oleic, and caprylic acids. These compounds have been associated with antioxidant and anti-inflammatory properties and may contribute to reducing the formation of reactive oxygen species and preserving the integrity of cellular membranes (Santos Souza et al. 2021; Barbosa et al. 2024; Sales et al. 2023). 

Furthermore, the improvement observed in pulmonary oxidative stress parameters may be related to the reduction in the systemic inflammatory response previously observed in this study, evidenced by decreased levels of IL-1β, IL-6, and TNF-α in the groups treated with higher doses of the oil. The interaction between inflammation and oxidative stress is widely recognized in pancreatitis, as inflammatory mediators can stimulate the production of free radicals and amplify tissue damage (Xia et al. 2024; Chen et al. 2025).

Although these molecular pathways were not directly investigated in the present study, the reduction in lipid peroxidation associated with increased SOD and CAT activity suggests that ScFO may modulate redox-sensitive signaling pathways involved in experimental pancreatitis. Taken together, these results suggest that ScFO may contribute to the modulation of pulmonary oxidative stress associated with experimental pancreatitis, reinforcing the protective effects observed in the other parameters evaluated in this study.

## Conclusion

ScFO demonstrated a protective effect in the experimental model of L-arginine–induced pancreatitis, particularly at doses of 50 mg/kg and 100 mg/kg. Treatment reduced markers of pancreatic injury, pro-inflammatory cytokines, and oxidative stress, in addition to attenuating histological alterations in lung tissue. These findings suggest that the oil may modulate inflammatory and oxidative processes associated with experimental pancreatitis, possibly related to the presence of bioactive fatty acids in its chemical composition.

## Data Availability

No datasets were generated or analysed during the current study.
